# Traumatic Carotid Artery Dissection Following a Brazilian Jiu-Jitsu Chokehold: A Case Report

**DOI:** 10.7759/cureus.91604

**Published:** 2025-09-04

**Authors:** Patricia Martinet, Olivia G Holloway, Constantine C Phatouros, William Wallefeld, David Prentice

**Affiliations:** 1 Stroke Rehabilitation, Perron Institute for Neurological and Translational Science, Perth, AUS; 2 Medicine, University of Western Australia, Perth, AUS; 3 Neurological Intervention and Imaging Service of Western Australia (NIISWA), Sir Charles Gairdner Hospital, Perth, AUS; 4 Neurology, Sir Charles Gairdner Hospital, Perth, AUS; 5 Neurosciences, Perron Institute for Neurological and Translational Science, Perth, AUS

**Keywords:** carotid artery dissection, cervical trauma, ischemia, non-fatal strangulation, stroke, vascular trauma

## Abstract

Carotid artery dissection is a rare but important cause of stroke in young adults, often resulting from trauma rather than atherosclerotic disease. This case describes a previously healthy 37-year-old male who experienced a sudden onset of left facial and arm numbness and weakness during exercise, shortly after participating in a Brazilian Jiu-Jitsu (BJJ) session. A chokehold maneuver one week earlier was identified as a likely precipitating factor. Initial symptoms resolved within an hour, but imaging a week later revealed a right internal carotid artery dissection with a small cortical infarct in the right insula. He was treated conservatively with aspirin, and follow-up imaging showed complete vessel healing. This case highlights the delayed and subtle presentation of carotid artery dissection following neck trauma in grappling sports. The mechanism typically involves vascular compression and neck hyperextension, resulting in intramural hematoma and potential thromboembolism. Timely diagnosis through detailed history-taking and vascular imaging is crucial to prevent stroke. Additionally, similar vascular injuries can result from non-fatal strangulation in assault or domestic violence, which may go unrecognized due to absent external signs. Clinicians should maintain a high index of suspicion for carotid dissection in young patients with acute neurological symptoms and recent neck trauma, regardless of mechanism.

## Introduction

Stroke in young adults often has atypical causes, with arterial dissection representing a leading etiology in this population [1]. While commonly spontaneous, dissections can also result from trauma, sometimes seemingly minor or occurring with a delay between injury and symptom onset. In contact and grappling sports like Brazilian Jiu-Jitsu (BJJ), chokeholds and neck manipulations have emerged as underrecognized mechanisms of vascular injury. A survey of 234 BJJ schools found that 28% of injuries involve the neck, underscoring its vulnerability [2]. Another study involving over 4,000 grapplers, primarily males between the ages of 18 and 44, evaluated the safety of sportive chokeholds. Many reported frequent choking experiences, with 75.7% experiencing pre-syncope symptoms and 27.8% losing consciousness at least once [3].

The mechanism of a chokehold injury typically involves a combination of direct compression of the carotid artery, creating a point of fixation, followed by hyperextension and rotation of the neck [4]. Carotid artery dissection results from a tear in the intimal layer of the arterial wall, allowing blood to enter and form an intramural hematoma. This can give rise to two primary pathological processes. The first involves artery-to-artery embolism, where a thrombus forms along the intimal tear and embolizing to distal sites [1,5]. The second mechanism involves luminal narrowing and flow obstruction, in which blood trapped between the tunica intima and tunica media reduces the vessel diameter, causing stenosis and potentially compressing nearby structures [1,5]. These processes help explain the typical delay between the initial trauma and the onset of ischemic symptoms. The anatomical relationship between structures like the stylohyoid process, vertebral transverse processes, or the hyoid bone can play a significant role in these injuries. For instance, if the hyoid bone is abnormally positioned, it can compress or irritate the carotid artery, potentially leading to dissection or aneurysm formation [6].

We present a case involving a healthy 37-year-old male who developed transient neurological symptoms one week after a BJJ session chokehold, with subsequent imaging revealing a carotid artery dissection. This case draws attention to the delayed and subtle presentation of traumatic vascular injury, the unique biomechanical forces involved in chokeholds, and the importance of detailed clinical history in young patients presenting with focal neurological deficits. The discussion also extends to other contexts where neck trauma may go unnoticed or underreported, such as in non-fatal strangulation associated with interpersonal violence.

## Case presentation

A 37-year-old right-handed male presented to the emergency department with the sudden onset of left-sided facial and arm numbness and weakness, which began while performing jumping exercises during Jiu-Jitsu training. He was examined at the scene by a doctor and found to have left facial droop and left handgrip weakness. By the time he arrived at the emergency department, about 45 minutes later, his symptoms had largely resolved. Seven days prior to the incident, the patient was sparring with a teammate when he was placed in a chokehold known as the “rear naked choke.” He did not lose consciousness or develop any further symptoms but did feel “unusual” for a short period of time afterward.

On review in the emergency department, vital signs were within normal limits. Neurological examination was unremarkable with no visual field defect and no motor-sensory deficit. The patient has a history of anxiety and a family history of heart disease in an uncle. He takes no regular medications. He is a non-smoker, rarely drinks alcohol and takes no recreational drugs.

**Figure 1:**
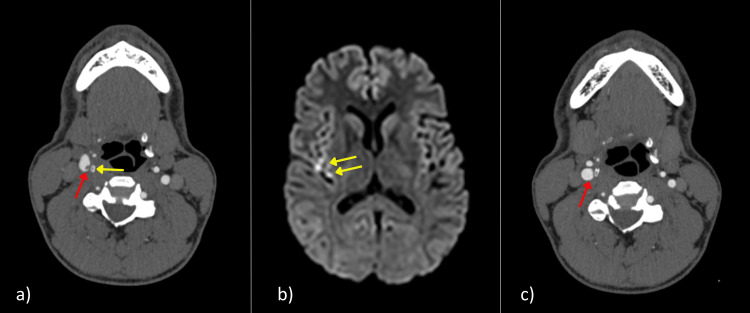
Imaging features of right internal carotid artery dissection in a 37-year-old male following a Brazilian Jiu-Jitsu chokehold. a) CT scan of the neck at presentation demonstrates an acute dissection flap of the medial wall of the right internal carotid artery (red arrow) immediately adjacent to the superior tip of the right greater cornu of the hyoid bone (yellow arrow). b) MRI diffusion-weighted imaging (DWI) sequence demonstrates a small area of acute ischemia infarction involving the right posterior insular cortex (yellow arrows). c) CT scan of the neck performed eight weeks after presentation shows remodeling of the small intimal dissection flap with resolution and return to normal appearance (red arrow).

As part of the emergency department workup, a non-contrast computed tomography (CT) scan of the brain was performed and was reported as normal. Subsequently, a CT angiogram (CTA) from the aortic arch to the circle of Willis was obtained. This demonstrated a linear filling defect along the posterior wall of the right proximal internal carotid artery, just distal to the bifurcation (Figure 1a), consistent with a small intimal dissection with an adherent thrombus. Magnetic resonance imaging (MRI) showed a small cortical-gyriform area of mild diffusion restriction with fluid-attenuated inversion recovery (FLAIR) and T2 hyperintensity in the posterosuperior aspect of the right insula, in keeping with a recent infarct (Figure 1b). The patient was commenced on aspirin and advised to avoid strenuous exercise. At eight weeks, a repeat CT angiogram demonstrated healed vessels, after which aspirin was discontinued (Figure 1c).

## Discussion

The case discussed highlights the range of symptoms and the delayed onset that can occur from carotid artery dissection following a chokehold. In a similar case, a 27-year-old man sustained a cervical carotid dissection while practicing a chokehold maneuver in BJJ. Initially, he noticed severe neck pain, and one week later, he had a complete left middle cerebral artery stroke requiring a craniectomy, leaving him with a severe neurological deficit [7]. Taekwondo, karate, judo, and mixed martial arts have all been associated with carotid dissection, some due to direct trauma from kicks or punches [7-9]. Symptoms can vary widely, ranging from mild and transient to severe and persistent, including headache, neck pain, cranial nerve palsies, pulsatile tinnitus, ataxia, and dizziness [10]. The delay between the traumatic event and symptom onset, which can range from hours to days, is a key feature. Following the carotid injury, whether from dissection or endothelial damage (both of which may be undetectable on a CT coronary angiogram), local thrombosis can form and embolize to the brain, resulting in a stroke. The initial event may be a transient ischemic attack, but as embolization continues, it can result in a devastating stroke syndrome or even death due to stroke-related cerebral edema. As there may be no initial symptoms, taking a detailed history, including recent participation in such sports, can provide a vital clue to the etiology.

While vascular risk factors such as coronary heart disease, hypertension, hypercholesterolemia, smoking, and migraines are commonly associated with spontaneous dissection, they are typically absent in younger patients experiencing traumatic carotid artery dissection. In these cases, genetic predispositions play a crucial role, particularly in individuals with connective tissue disorders like Ehlers-Danlos syndrome and Marfan syndrome, which confer an intrinsic susceptibility to vascular complications [11].

The anatomical proximity and relationship of surrounding structures are thought to increase the vulnerability of the carotid artery. Notably, the distance between the styloid process and the internal carotid artery has been identified as a potential risk factor for carotid artery dissection [12,13]. This mechanism is believed to involve repeated microtrauma to the vessel wall caused by routine head and neck movements, with the risk being particularly pronounced in individuals with inherently weakened connective tissue [12].

Chokeholds and neck compression are also a feature of non-fatal strangulation occurring in cases of domestic violence and assault. In a study of 1,064 women referred to a Sexual Assault Resource Centre (SARC), 7.4% (79 women) alleged that a non-fatal strangulation occurred during the assault [14]. Disturbingly, the odds of non-fatal strangulation were eight times higher when perpetrated by the victim's intimate partner. The incidence of carotid injury in manually strangled patients is variable but likely low. A retrospective study found two arterial dissections in 328 patients (0.6%), but only 57% of non-fatal strangulation cases were imaged [15]. Notably, approximately 50% of patients in non-fatal strangulation cases show no visible physical injuries [14,16]. This highlights the importance of recognizing strangulation as a potential cause of idiopathic carotid dissection, particularly as such incidents are often concealed in cases of domestic abuse. For example, a 43-year-old woman with a middle cerebral artery stroke and symmetrical internal carotid stenoses reported that her husband had attempted to strangle her three months earlier [17]. It is also conceivable that strangulation damages the ligaments and fascia in the neck, enabling carotid mobility and impingement syndromes. These studies highlight the importance of considering non-fatal strangulation in various contexts beyond martial arts, particularly in domestic violence and assault cases. In cases of spontaneous or sudden neurological deficits in younger adults without prior medical history, it is crucial to obtain a detailed history of any previous violence, including strangulation, even if it was a single incident. Manual strangulation should be considered in the differential diagnosis of stroke in young patients. Emergency physicians should also be aware that internal injuries may take hours to manifest, and the patient may be at risk of delayed fatal outcomes.

Due to the risk of vascular injury from non-fatal strangulation chokeholds, a thorough diagnostic approach is crucial when cervical artery dissection is suspected. The initial diagnosis can be established through a combination of duplex ultrasound and CT angiography [5]. However, if clinical suspicion remains high despite initial negative findings, formal carotid angiography may be warranted for definitive assessment. In cases where vascular injury is confirmed, MRI brain imaging is essential to assess the extent, volume, and distribution of any resultant infarction. Clinical decision-making becomes even more complex in pregnant patients presenting to the emergency department after a non-fatal chokehold. Airway injuries can be evaluated using nasendoscopy, CT, or a combination of both. Nasendoscopy is useful for assessing and documenting bruising and swelling in the airways.

The Biffl grading system classifies blunt cerebrovascular injuries (BCVI) of the carotid and vertebral arteries into five grades [18]. Grade I represents a minor intimal injury or luminal irregularity with less than 25% narrowing. Grade II refers to dissections or intramural hematomas that cause more than 25% narrowing, raised intimal flaps, or intraluminal thrombus. Grade III is defined by pseudoaneurysm formation, while Grade IV indicates complete vessel occlusion. Grade V describes vessel transection with active extravasation or bleeding. This framework provides a structured link between injury pattern, prognosis, and management: Grades I and II are typically treated with antithrombotic therapy, whereas higher-grade injuries involving pseudoaneurysms, occlusions, or transections may require surgical or endovascular repair. Prognostically, stroke risk increases with higher Biffl grades in carotid artery injuries, while the relationship is less predictable in vertebral artery injuries due to the presence of collateral posterior circulation [18].

According to current evidence, blunt traumatic carotid and vertebral artery dissections are most often managed conservatively with individualized antithrombotic therapy (either antiplatelet or anticoagulation, depending on risk profile), close clinical monitoring, and repeat vascular imaging, typically CTA/magnetic resonance angiography (MRA) at approximately six weeks to evaluate vessel healing [5]. In the Biffl system, endovascular repair (such as stenting or coiling) may be indicated for Grade III pseudoaneurysms, thrombectomy is reserved for acute ischemic stroke complicating Grade IV occlusions, and Grade V transections with active hemorrhage typically require urgent surgical or endovascular intervention [5,19]. Traditionally, a thrombectomy procedure was limited to patients who presented within six hours of stroke onset. However, data now indicates a procedural benefit to some patients as much as 24 hours after a stroke. The decision to proceed with thrombectomy is now guided by individual patient factors, including the type of vessel occlusion (large, medium, or small), stroke severity, and the amount of salvageable brain tissue [19].

Thorough history-taking is crucial in cases of carotid artery dissection, as it can reveal key causes such as grappling sports injuries or non-fatal strangulation in domestic violence. Chokeholds and neck trauma, common in sports like Brazilian jiu-jitsu, significantly increase the risk of dissection, often with delayed symptom onset ranging from headaches to severe strokes. The initial two weeks are regarded as the period of greatest risk for stroke following both traumatic and spontaneous dissections [20]. Conditions like Ehlers-Danlos or Marfan syndrome and anatomical vulnerabilities further heighten risk, noting that such vulnerabilities may remain clinically unknown. Clinicians must consider both physical trauma and social contexts to ensure timely and accurate diagnosis.

## Conclusions

This case underscores the importance of recognizing carotid artery dissection as a potential cause of stroke in young, otherwise healthy individuals following neck trauma. Chokeholds in grappling sports such as Brazilian Jiu-Jitsu represent a significant yet often underappreciated risk factor, particularly given the possibility of delayed symptom onset and subtle early signs. A thorough clinical history, including participation in high-risk activities or experiences of non-fatal strangulation, is vital to prompt diagnosis and management. Early imaging and appropriate treatment can prevent serious complications, including permanent neurological deficits or death. Greater awareness among clinicians, especially in emergency and primary care settings, is essential to improving outcomes in patients with trauma-related vascular injuries.

## References

[1] Malhotra K, Goyal N, Tsivgoulis G (2017). Internal carotid artery occlusion: pathophysiology, diagnosis, and management. Curr Atheroscler Rep.

[2] Hunker JJ, Tarpada SP, Khoury J, Goch A, Kahn M (2023). Injuries common to the Brazilian Jiu-Jitsu practitioner. Cureus.

[3] Stellpflug SJ, Schindler BR, Corry JJ, Menton TR, LeFevere RC (2020). The safety of sportive chokes: a cross-sectional survey-based study. Phys Sportsmed.

[4] Deen R, Austin C, Bullen A (2023). Review article: Non-penetrating neck artery dissection in young adults: not to be missed!. Emerg Med Australas.

[5] Yaghi S, Engelter S, Del Brutto VJ (2024). Treatment and outcomes of cervical artery dissection in adults: a scientific statement from the American Heart Association. Stroke.

[6] Ye Q, Liu Y, Tang H, Zhou Q, Zhang H, Liu H (2023). Hyoid bone compression‑induced carotid dissecting aneurysm: a case report. Exp Ther Med.

[7] Demartini Z Jr, Rodrigues Freire M, Lages RO, Francisco AN, Nanni F, Maranha Gatto LA, Koppe GL (2017). Internal carotid artery dissection in Brazilian Jiu-Jitsu. J Cerebrovasc Endovasc Neurosurg.

[8] Stellpflug SJ, Dummer MF, Martin CD, Vera JA, LeFevere RC (2022). Cervical artery dissections and ischemic strokes associated with vascular neck compression techniques (sportive chokes). J Emerg Med.

[9] Schlemm L, Nolte CH, Engelter ST, Endres M, Ebinger M (2017). Cervical artery dissection after sports: an analytical evaluation of 190 published cases. Eur Stroke J.

[10] Singh RR, Barry MC, Ireland A, Bouchier Hayes D (2004). Current diagnosis and management of blunt internal carotid artery injury. Eur J Vasc Endovasc Surg.

[11] Gunduz ME, Kadirvel R, Kallmes DF, Pezzini A, Keser Z (2023). Spontaneous cervical artery dissection: is it really a connective tissue disease? A comprehensive review. Front Neurol.

[12] Venturini G, Vuolo L, Pracucci G, Picchioni A, Failli Y, Benvenuti F, Sarti C (2023). The proximity between styloid process and internal carotid artery as a possible risk factor for dissection: a case-control study. Neuroradiology.

[13] Amorim JM, Pereira D, Rodrigues MG (2018). Anatomical characteristics of the styloid process in internal carotid artery dissection: case-control study. Int J Stroke.

[14] Zilkens RR, Phillips MA, Kelly MC, Mukhtar SA, Semmens JB, Smith DA (2016). Non-fatal strangulation in sexual assault: a study of clinical and assault characteristics highlighting the role of intimate partner violence. J Forensic Leg Med.

[15] Matusz EC, Schaffer JT, Bachmeier BA (2020). Evaluation of nonfatal strangulation in alert adults. Ann Emerg Med.

[16] White C, Martin G, Schofield AM, Majeed-Ariss R (2021). 'I thought he was going to kill me': analysis of 204 case files of adults reporting non-fatal strangulation as part of a sexual assault over a 3 year period. J Forensic Leg Med.

[17] Malek AM, Higashida RT, Phatouros CC, Halbach VV (1999). A strangled wife. Lancet.

[18] Biffl WL, Moore EE, Offner PJ, Burch JM (2001). Blunt carotid and vertebral arterial injuries. World J Surg.

[19] Nogueira RG, Jadhav AP, Haussen DC (2018). Thrombectomy 6 to 24 hours after stroke with a mismatch between deficit and infarct. N Engl J Med.

[20] Morris NA, Merkler AE, Gialdini G, Kamel H (2017). Timing of incident stroke risk after cervical artery dissection presenting without ischemia. Stroke.

